# A thermostable organic solvent-tolerant lipase from *Brevibacillus* sp.: production and integrated downstream processing using an alcohol-salt-based aqueous two-phase system

**DOI:** 10.3389/fmicb.2023.1270270

**Published:** 2023-10-13

**Authors:** Senaite Leykun, Eva Johansson, Ramesh Raju Vetukuri, Elaine Berger Ceresino, Amare Gessesse

**Affiliations:** ^1^Institute of Biotechnology, Addis Ababa University, Addis Ababa, Ethiopia; ^2^Department of Biological Sciences and Biotechnology, Botswana International University of Science and Technology, Palapye, Botswana; ^3^Department of Plant Breeding, Swedish University of Agricultural Sciences, Lomma, Sweden

**Keywords:** thermostable lipase, *Brevibacillus*, solvent-tolerant lipase, lipase immobilization, transesterification reaction, aqueous two-phase system

## Abstract

Lipases are used for the synthesis of different compounds in the chemical, pharmaceutical, and food industries. Most of the reactions are carried out in non-aqueous media and often at elevated temperature, requiring the use of organic solvent-tolerant thermostable lipases. However, most known lipases are not stable in the presence of organic solvents and at elevated temperature. In this study, an organic solvent-tolerant thermostable lipase was obtained from *Brevibacillus* sp. SHI-160, a moderate thermophile isolated from a hot spring in the East African Rift Valley. The enzyme was optimally active at 65°C and retained over 90% of its activity after 1 h of incubation at 70°C. High lipase activity was measured in the pH range of 6.5 to 9.0 with an optimum pH of 8.5. The enzyme was stable in the presence of both polar and non-polar organic solvents. The stability of the enzyme in the presence of polar organic solvents allowed the development of an efficient downstream processing using an alcohol-salt-based aqueous two-phase system (ATPS). Thus, in the presence of 2% salt, over 98% of the enzyme partitioned to the alcohol phase. The ATPS-recovered enzyme was directly immobilized on a solid support through adsorption and successfully used to catalyze a transesterification reaction between paranitrophenyl palmitate and short-chain alcohols in non-aqueous media. This shows the potential of lipase SHI-160 to catalyze reactions in non-aqueous media for the synthesis of valuable compounds. The integrated approach developed for enzyme production and cheap and efficient downstream processing using ATPS could allow a significant reduction in enzyme production costs. The results also show the potential of extreme environments in the East African Rift Valley as sources of valuable microbial genetic resources for the isolation of novel lipases and other industrially important enzymes.

## 1. Introduction

Application of lipases in the food, fine chemical, pharmaceutical, olio-chemical, biofuel, cosmetics, detergent, and leather tanning industries attracted the attention of researchers for the development of novel lipases suited for such varied applications (Filho et al., 2019; Chandra et al., 2020; Akram et al., 2023; Ali et al., 2023). In nature, lipases are hydrolytic enzymes involved in the breakdown of triglycerides in aqueous media by acting at the hydrophobic–hydrophilic interphase. However, most reactions involving industrial applications of lipases are carried out in non-aqueous media where water is replaced by different organic solvents. In addition, some reactions are carried out at elevated temperature (Hasan et al., 2006; Salihu and Alam, 2015; Nezhad et al., 2022). This is because catalysis in non-aqueous media offers several advantages, such as increased solubility of hydrophobic substrates in the reaction media, better substrate and product stability, and favoring synthesis over hydrolysis (Cao and Matsuda, 2016; Ramos-Martín et al., 2020). However, exposure to organic solvents and/or elevated temperature could pose challenges to enzyme activity and stability (Kumar et al., 2016; Kikani et al., 2023). Therefore, the use of organic solvent-tolerant thermostable lipases to catalyze reactions in non-aqueous media offers several biotechnological advantages (Ismail et al., 2021; Haryati et al., 2022; Kikani et al., 2023). One approach to develop thermostable enzymes involves isolation and screening of microbes from naturally occurring high-temperature environments, such as hot springs, that harbor a diverse group of microorganisms. These microorganisms are physiologically adapted to grow and multiply in the moderate-to-high temperature range, and their cellular machineries, including enzymes, are optimally active and stable at elevated temperature. To date, lipase-producing thermophilic and moderately thermophilic microorganisms were reported from different laboratories (Vivek et al., 2022), some of them producing organic solvent-tolerant enzymes. These include *Nocardiopsis* sp. (Aziz et al., 2020), *Bacillus* sp. (Nomwesigwa et al., 2023), *Bacillus stearothermophilus* (Bacha et al., 2015), *Bacillus licheniformis* (Sharma and Kanwar, 2017), *Pseudomonas fluorescens*, (Hu et al., 2023), *Bacillus thermoleovorans* (Abol-Fotouh et al., 2016), *Aneurinibacillus* sp. (Masomian et al., 2010), *Burkholderia multivorans* (Boran and Ugur, 2015), *Bacillus subtilis* (Rathi et al., 2015), *Bacillus coagulans* (Lianghua and Liming, 2005), *Geobacillus thermodenitrificans* (Christopher et al., 2015), and *Streptomyces* sp. (Mander et al., 2012). However, several other thermophilic microbial genera are known to exist in different high-temperature environments, one of which is the member of the genus *Brevibacillus*. Although some strains belonging to the genus *Brevibacillus* were reported to produce some industrially important enzymes, such as proteases (Rai and Mukherjee, 2011) and α-amylases (Suribabu et al., 2014), to date, there are very few reports on lipase production by thermophilic strains of the genus *Brevibacillus* (Panda et al., 2015; Atanasova et al., 2023).

The Great East African Rift Valley is a geologically active region with several extreme environments, such as neutral and alkaline hot springs, with temperature ranging from medium to high, soda lakes with pH up to 10.5, and habitats with medium to high salinity. Isolation of lipase-producing thermophilic microbial strains from these habitats is expected to provide microbial strains, producing novel lipases. However, to date, no thermostable lipase-producing microbial strain has been reported from these habitats. The objective of the present study was to isolate lipase-producing thermophilic or moderately thermophilic strains from Shala hot spring, characterize the enzyme to determine its potential industrial applications, develop a cost-effective downstream processing for enzyme recovery using an alcohol-salt-based aqueous two-phase system, and evaluate the potential of the enzyme to catalyze reactions in non-aqueous media.

## 2. Materials and methods

### 2.1. Enrichment, isolation, and screening for lipase production

Water and sediment samples were collected from Shala hot spring (07° 28' 677” N and 038° 38' 102” E) located at the shore of Lake Shala in the central Great Rift Valley of Ethiopia. During sample collection, water and sediment samples were collected along the course of the hot spring starting from the underground discharge point to the spring water that joins the lake water. In addition, a microbial mat sample with a water temperature of ~45°C was collected from underneath the flowing water near the discharge point of the hot spring. All samples were transferred to sterile 50-ml screw-capped vials and transported to the laboratory in an icebox kept at 4°C. Temperature, pH, and conductivity of the hot spring were measured on-site during sampling.

Sediment samples were suspended in a phosphate buffer saline solution, and the supernatant was used as an inoculum. To prepare an enrichment culture, 1 ml of the hot spring water sample or 1 ml of the sediment suspension was inoculated into a 100 ml enrichment medium in a 500 ml flask and incubated at 55°C for 48 h with rotary shaking at 120 rpm. The culture medium used for enrichment was composed of (g/l) olive oil, 10; yeast extract, 2.5; peptone, 2.5; NaCl, 5; CaCl_2_.2H_2_O, 0.1; and MgSO_4_.7H_2_O, 0.1. The medium was then homogenized using an overhead blender until the solution became milky. After autoclaving at 121°C and 15 psi, the pH of the medium was adjusted to 8.5 by adding sterile concentrated sodium carbonate bicarbonate buffer. Solid medium was prepared for isolation by adding 15 g/l agar to the medium used for enrichment.

A sample of the enrichment culture was serially diluted using a sterile saline solution, and 100 μl was spread on agar plates. After 48 h of incubation at 55°C, colonies were transferred to fresh agar plates, purified through repeated streaking, and kept at 4°C for further screening.

Initial screening for lipase production was carried out on agar plates containing the same composition as the isolation media and growth condition at 55°C, except that Tween 80 was replaced with olive oil. Isolates forming a white zone of a calcium-free fatty acid precipitate were selected as lipase-positive and taken for further screening through a liquid medium containing olive oil in place of Tween 80.

### 2.2. Enzyme production in liquid culture

All positive isolates were grown in a 500 ml flask containing (g/l) olive oil, 3; peptone, 3; yeast extract, 3; CaCl_2_.2H_2_O, 0.1; MgSO_4_.7H*2*O, 0.1; and NaCl, 5. The medium was inoculated with fresh 12-h old culture and allowed to grow at 55°C for 18 h with rotary shaking at 120 rpm. The cell-free culture supernatant was used as the crude enzyme source.

### 2.3. Lipase activity assay

Lipase activity was measured using paranitrophenyl palmitate (pNPP) as a substrate, with slight modification according to Mahadik et al. (2002). A 1 mM substrate was prepared from a 20 mM stock solution of pNPP in isopropanol in 20 mM Tris-HCl buffer containing 0.1% gum arabic and 0.4% Triton X-100. The enzyme reaction was initiated by mixing 0.9 ml of the above substrate solution with 0.1 ml of the enzyme. After 10 min of incubation at 60°C, the reaction mixture was transferred to an ice bath, followed by measurement of the absorbance of the resulting colored compound at 410 nm against a reagent blank.

The amount of pNP released was calculated using the apparent extinction coefficient of 14,800 M^−1^cm^−1^ under the assay condition. One unit of lipase activity was defined as the amount of enzyme that releases 1 nmol of the product per minute under the assay condition.

### 2.4. Optimization of cultivation conditions

#### 2.4.1. Effect of nitrogen and carbon source

To study the effect of media components on lipase production, a basal medium supplemented with different nitrogen and carbon sources was used. The basal medium was composed of (g/l) NaCl, 5; CaCl_2_.2H_2_O, 0.1; and MgSO_4_.7H_2_O, 0.1. After sterilization, pH was adjusted to 8.5 using 0.5M sodium carbonate bicarbonate buffer. To test the effect of nitrogen sources, the basal medium containing 1% olive oil was supplemented with different concentrations (0, 0.25, 0.5, and 1%) of the nitrogen source. The nitrogen sources tested were peptone, yeast extract, and ammonium nitrate. After selection of the best nitrogen source, the effect of different carbon sources was tested in the presence of 0.5% of different carbon sources (glucose, glycerol, xylose, starch, lactose, or sucrose), with the basal medium supplemented with 0.25% yeast extract.

Similarly, to evaluate the effect of inducers, the basal medium containing 0.25% yeast extract was supplemented with 0.5% each of olive oil, Tween 80, and sunflower oil. The medium was inoculated with a 2% inoculum from a 10-h culture and incubated at 55°C with shaking at 120 rpm. After 18 h growth, the culture was harvested by centrifugation at 10,000 rpm for 10 min, and the cell-free culture supernatant was used as the enzyme source.

#### 2.4.2. Effect of culture medium pH on lipase production

To test the effect of pH on lipase production, the organism was grown using the basal medium containing 0.25% yeast extract and 0.5% glucose, and then the pH was adjusted from 7.0 to 10 with an interval of 0.5 pH. After inoculation with 2% inoculum, the culture was incubated at 55°C with rotary shaking. Lipase activity was measured from the cell-free culture supernatant and recovered from the culture after 18 h growth.

#### 2.4.3. Time course of lipase production

To determine the optimum time for growth and enzyme production, the organism was grown in the basal medium containing 0.5% glucose as a carbon source and 0.25 % yeast extract as a nitrogen source at 55°C and pH 8. To determine lipase production, the cells were separated by centrifugation, and the cell-free supernatant was used as the enzyme source.

### 2.5. Molecular identification of the selected isolate

Bacterial DNA was isolated using Quick-DNA™ Fungal/Bacterial Miniprep Plus Zymo Research Kit according to the manufacturer's instruction. NanoDrop Micro Photometer (NanoDrop Technologies, UK) and agarose gel electrophoresis were used to determine the yield and integrity of the DNA, respectively. The following universal primer pairs were used to amplify the 16s rRNA region of the bacterial isolate: 27F (5′-AGAGTTTGATCMTGGCTCAG-3′) (Lane, 1991) and 907R (5′-CCGTCAATTCMTTTRAGTTT-3′) (Morales and Holben, 2009). PCR reactions were performed with 10 ng of DNA using the following temperature parameters: an initial denaturation phase at 94°C for 3 min, followed by 35 cycles at 94°C for 45 s, 50°C for 30 s, and 72°C for 30 s, followed by a final extension step at 72°C for 5 min. The Qiagen QIAquick PCR Purification Kit was used to purify the PCR products (Qiagen, UK). Sanger sequencing for species identification was performed at the Eurofins Genomics sequencing facility (Germany) using 27F and 907R primers. SnapGene was used (SnapGene, USA) to analyze the sequences obtained manually from the sequencing platform. The resulting sequences including the 16 s region were compared with the National Center for Biotechnology Information (NCBI) GenBank non-redundant nucleotide database [BLASTn (Altschul et al., 1997)]. The coverage and identity of search matches to sequences from database entries were examined, and the best-matched NCBI accession was reported. The microorganism was determined as *Brevibacillus* sp. SHI-160, with GenBank accession number ON646459.

### 2.6. Purification and characterization of *Brevibacillus* sp. SHI-160 lipase

#### 2.6.1. Enzyme purification

The crude enzyme preparation was precipitated by adding solid ammonium sulfate to 70% saturation with continuous stirring at 4°C. The precipitate was centrifuged at 10,000 rpm for 30 min at 4°C, and the pellet was resuspended in 10 mM Tris-HCl buffer of pH 8 and dialyzed against two changes of the same buffer.

The dialyzed enzyme preparation was applied to a DEAE cellulose column equilibrated with 10 mM Tris-HCl buffer. The column was washed with eight column volumes of 0.5 M NaCl in 10 mM Tris-HCl buffer to remove other bound proteins. The lipase was then eluted using 0.8 M NaCl containing 0.015% Triton X-100 in 10 mM Tris-HCl buffer. Fractions with lipase activity were pooled and dialyzed with two changes of 2 L of 50 mM Tris-HCl buffer, pH 8. The purified protein was lyophilized and resuspended in a minimum volume of buffer for further purity testing in SDS-PAGE.

#### 2.6.2. SDS-PAGE and zymography

The purity of lipase was checked by SDS-PAGE analysis using 15 % resolving gel and 4.5 % stacking gel. Protein concentration was determined using the Lowry method (Lowry et al., 1951).

For zymographic detection of lipase activity, 5 μl of the enzyme sample was mixed with sample buffer and run using a 15% SDS-PAGE gel. To remove the SDS, the gel was washed using 1% Triton X-100 in 25 mM Tris-HCl buffer, pH 8, followed by washing and equilibration with 25 mM Tris-HCl buffer (Kwon et al., 2011). The washed gel was then overlayed with a second freshly prepared gel that contained 0.5% Tween 80 and 0.01% CaCl_2._ After 10 min at 50°C, a white band formed due to the formation of a precipitate that resulted from a reaction between fatty acids released from the hydrolysis of Tween 80, and the calcium ion is used as an indicator of the presence of lipase.

#### 2.6.3. Effect of temperature on activity and stability of SHI-160 lipase

To determine the optimum activity temperature of the SHI-160 lipase, the activity of the purified enzyme was assayed in the temperature range of 45–80°C in the presence and absence of 5 mM Ca^2+^.

To test the effect of temperature on stability, SHI-160 lipase was incubated at 60, 65, and 70°C for up to 3 h in the presence and absence of 5 mM CaCl_2_. Samples were withdrawn at 1-h intervals, the residual activity was assayed following a standard assay procedure, and the residual activity was calculated relative to the initial activity.

#### 2.6.4. Effect of pH on the activity and stability of SHI-160 lipase

The effect of pH on the activity was determined in the range of 3.5 to 10.5. The buffers used, each at 20 mM, were acetate (pH 3.5 to 5.5), phosphate (pH 5.5 to 8.0), Tris-HCl (7.5 to 9), and glycine-NaOH (9.0 to 10.5). To avoid the effect of pH change on the color intensity of the released paranitrophenyl, at the end of the reaction, the pH of all reaction mixtures was adjusted to 8.0 using 0.5 M Tris-HCl buffer before the absorbance was measured.

To test the effect of pH on stability, SHI-160 lipase was incubated at different pH values for 1 h at 37°C, followed by measurement of activity using the standard assay procedure. Residual activity was calculated as a percentage of the original activity.

#### 2.6.5. Effect of metal ion, inhibitors, and salt concentration on SHI-160 lipase

The enzyme was incubated for 1 h at 37°C in the presence of 1 and 5 mM of different metal ions (Mn^2+^, Co^2+^, Mg^2+^, Na^+^, Ca^2+^, Cu^2+^, Fe^2+^, K^+^, and Zn^2+^) and inhibitors (i.e., PMSF, EDTA, DTT, and SDS), followed by measurement of residual activity.

The effect of salt on lipase activity was assayed in the presence of 0.1 up to 2 M NaCl in 20 mM Tris-HCl buffer, pH 8.5. To control the effect of the high salt concentration on the substrate, for each salt concentration, independent reagent blanks were prepared.

#### 2.6.6. Effect of organic solvent on the stability of SHI-160 lipase

The effect of different organic solvents on the stability of lipase was determined by incubating the purified lipase in the presence and absence of different polar and non-polar organic solvents (acetone, ethanol, methanol, isopropanol, DMSO and acetonitrile, hexane, and ethyl acetate). Organic solvents were added to a final concentration of 25, 50, and 75% to the enzyme suspended in 50 mM Tris-HCl buffer and incubated for 1 h at 37°C. Throughout the incubation, the mixture was kept shaking at 200 rpm to increase the contact between the lipase and the solvent. At the end of the incubation period, the enzyme activity was assayed and expressed relative to the original activity.

### 2.7. Lipase extraction using alcohol-salt-based aqueous two-phase system

A binodal curve was prepared by mixing isopropanol and potassium phosphate solution following the turbidimetric titration method (Forciniti, 2000). To isolate the lipase directly from the culture, an 18-h old culture was mixed with the ATPS system by varying the concentration of phase-forming component. After complete mixing of all components using a vortex mixer, the tube was centrifuged for 1 min at 4,000 rpm. The volume of the top and bottom phases was recorded, and lipase activity and protein concentration were determined.

The efficiency of the process for lipase recovery was determined by calculating the different parameters shown in Supplementary Table 1. The optimum concentration of ATPS components was determined based on the yield of lipase activity recovered and the partition coefficient of the lipase. To evaluate the effect of salt on lipase extraction efficiency between 1 and 5% (w/w), NaCl was included in 16/16 (%w/w) of the isopropanol/potassium phosphate ATPS system.

#### 2.7.1. Direct adsorption of ATPS-recovered lipase on solid support

Lipase SHI-160 was extracted directly from 110 g of culture using a 16/16 % (w/w) isopropanol/phosphate ATPS in the presence of 2% NaCl (w/w). The enzyme was recovered from the top alcohol-rich phase and adsorbed onto celite 545^TM^. After removal of the solvent through drying at 45°C, lipase activity was measured using the standard pNPP assay. The efficiency of the formulation procedure was calculated using the following equation.


$$\begin{array}{c} \text{Yield }\left(\%\right)\text{=}\\ \text{ 100-}(\frac{\text{Activity of ATPS-recovered lipase}\left(\text{U}\right)\text{ - Activity of adsorbed lipase}\left(\text{U}\right)}{\text{Activity of ATPS-recovered lipase}\left(\text{U}\right)})\\ {}*100 \end{array}$$

#### 2.7.2. Evaluation of ATPS-recovered and immobilized lipase for transesterification reaction

Lipase SHI-160 recovered through ATPS directly from the culture supernatant and immobilized through adsorption was used to catalyze a transesterification reaction between pNPP and short-chain aliphatic alcohol as a substrate in non-aqueous media, following a previously reported procedure (Kotogán et al., 2014). In brief, a stock solution of 10 mM pNPP was prepared in an organic solvent (hexane or isooctane). The reactants were mixed to give a final concentration of 9 mM pNPP and 1.87 mM short-chain alcohol. The reaction was carried out for 30 min at 65°C. The transesterification activity of lipase was expressed in U/mg of lipase activity.

## 3. Result

### 3.1. Isolation and screening

Out of 543 bacterial isolates screened, 58 isolates (10.7%) were positive for extracellular lipase production on Tween 80 agar plates (Supplementary Figure 1). Lipase-positive isolates formed a white opaque zone around the colonies, indicating hydrolysis of the substrate. Upon further screening, 33 isolates (~6% out of the total screened) produced lipase in the liquid medium. Out of these, 12 isolates were selected based on the amount of lipase produced and subjected to further screening based on the thermostability of the enzyme each produced. One isolate, designated as SHI-160, produced a lipase that showed the highest thermostability at 70°C and was selected for further study. Based on the 16S rRNA gene sequence, isolate SHI-160 was identified as a strain of the genus *Brevibacillus*.

### 3.2. Optimization of production condition

#### 3.2.1. Effect of nitrogen and carbon sources on lipase production

Lipase production was highest (223.5 U/ml) when *Brevibacillus* sp. SHI-160 was grown using 0.25% yeast extract as the sole nitrogen source. However, lipase production significantly decreased when the concentration of yeast extract was increased to 0.5 and 1.0% (Table 1).

**Table 1:** Effect of nitrogen source at different concentrations on lipase production by *Brevibacillus* sp. SHI-160.

**Nitrogen sources (%)**		**Lipase production (U/ml)**
**Peptone/inorganic nitrogen**	**Yeast extract added**	
Peptone (0%)	0.25	223.5 ± 3.5
	0.5	113.8 ± 4.5
	1.0	62.4 ± 1.9
Peptone (0.25%)	0	22.0 ± 0.8
	0.25	185.0 ± 5.0
	0.5	154.5 ± 5.0
Peptone (0.5%)	0	151.4 ± 1.7
	0.25	106.3 ± 5.0
	0.5	144.8 ± 4.3
Peptone (1.0%)	0	10.3 ± 6.7
	0.25	91.4 ± 5.9
	0.5	103.1 ± 4.9
NH_4_NO_3_ (0.25%)	0	NG^*^
	0.25	207.9 ± 0-7

* NG, no growth. The organism was grown at 55°C, and the enzyme activity was determined from the cell-free culture supernatant collected after 18 h.

High lipase activity was also observed when 0.5% peptone was used as the sole nitrogen source but lower than the amount of enzyme produced when yeast extract was used as the sole nitrogen source (Table 1). The use of peptone and yeast extract as nitrogen sources in combination did not lead to an improvement in lipase production, as shown in Table 1. When grown using ammonium nitrate as the sole nitrogen source, no lipase production was observed because the organism failed to grow. However, enzyme production was observed when the organism was grown in the presence of ammonium nitrate and 0.25% yeast extract (Table 1), indicating the inability of the organism to utilize the inorganic nitrogen rather than inhibiting the growth or enzyme production.

**Figure 1:**
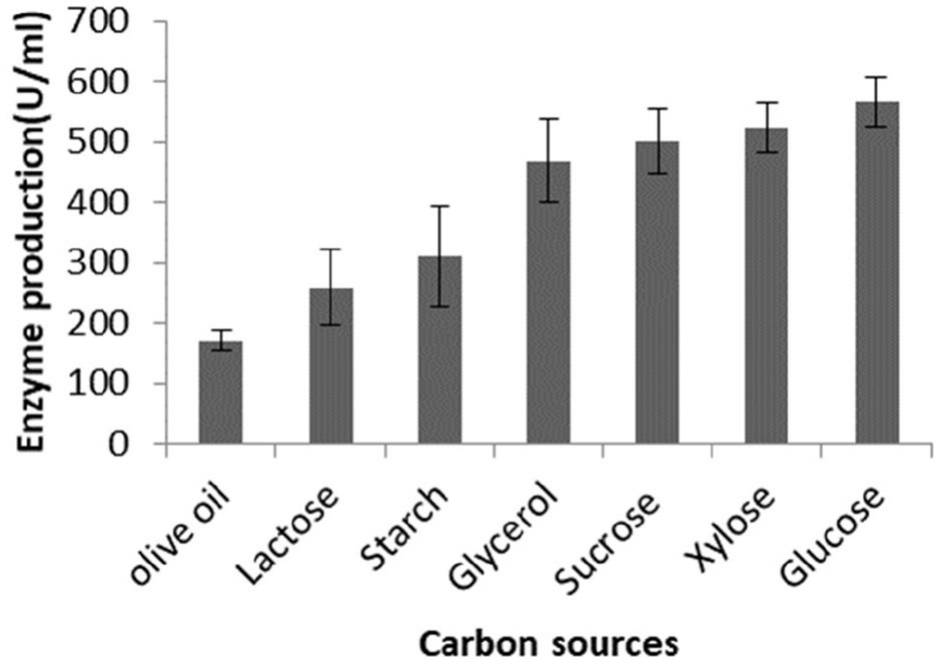
Effect of the addition of different carbon sources on lipase production by *Brevibacillus* sp. SHI-160. The organism was grown in a basal medium containing olive oil and 0.25% yeast extract. The medium was then supplemented with different sugars, and lipase production was measured from an 18-h culture.

To test the effect of different sugars on lipase production, *Brevibacillus* sp. SHI-160 was grown in a basic medium containing 0.5% olive oil and 0.25% yeast extract and supplemented with different sugars. Compared with the culture grown using olive oil as the sole carbon source, the addition of glycerol, sucrose, xylose, and glucose increased lipase production by up to 2-fold or more (Figure 1). For example, the addition of glucose increased lipase production by more than 3-fold (from 170 U/ml to 566 U/ml). Of all the sugars tested, the least amount of lipase production was observed in the presence of starch and lactose but still higher than when olive oil was used as the sole carbon source (Figure 1).

**Figure 2:**
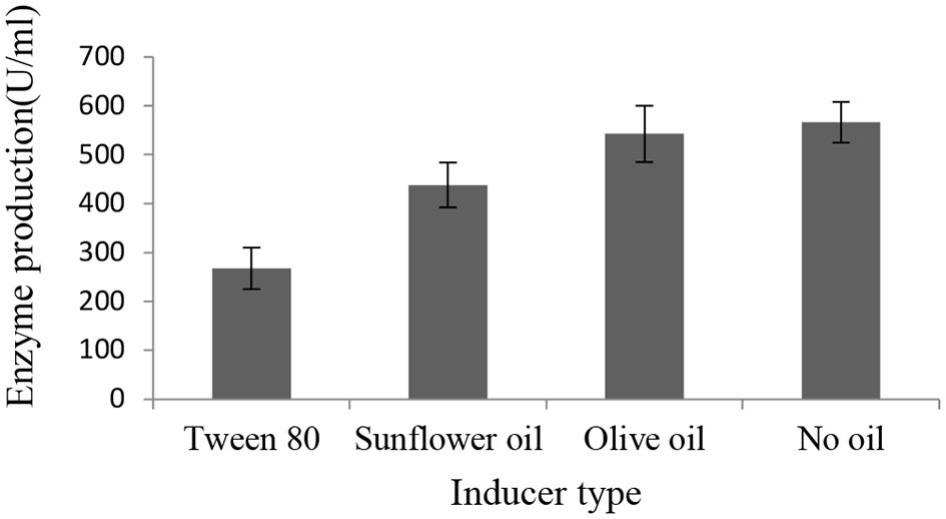
Effect of additional carbon source as an inducer on the level of SHI160 lipase production from *Brevibacillus* sp. SHI-160. The basal medium containing 0.25% yeast extract and 0.5% glucose was supplemented with 1% of lipid sources, and lipase production compared with the medium that contained no oil.

After we observed increased lipase production in the presence of sugars, we tested whether the isolate required lipid substrate as inducers. The results showed that *Brevibacillus* sp. SHI-160 did not require the addition of lipids as inducers for lipase production (Figure 2). When grown in the presence and absence of different inducers, the highest amount of lipase production was observed in the medium that contained 0.5% glucose as the sole carbon source. The addition of olive oil, sunflower oil, and Tween 80 did not result in an increase in enzyme production. When Tween 80 was added, growth was reduced by more than 50% (Supplementary Table 2), resulting in a much lower level of lipase production (Figure 2).

#### 3.2.2. Effect of medium pH on the level of lipase production

**Figure 3:**
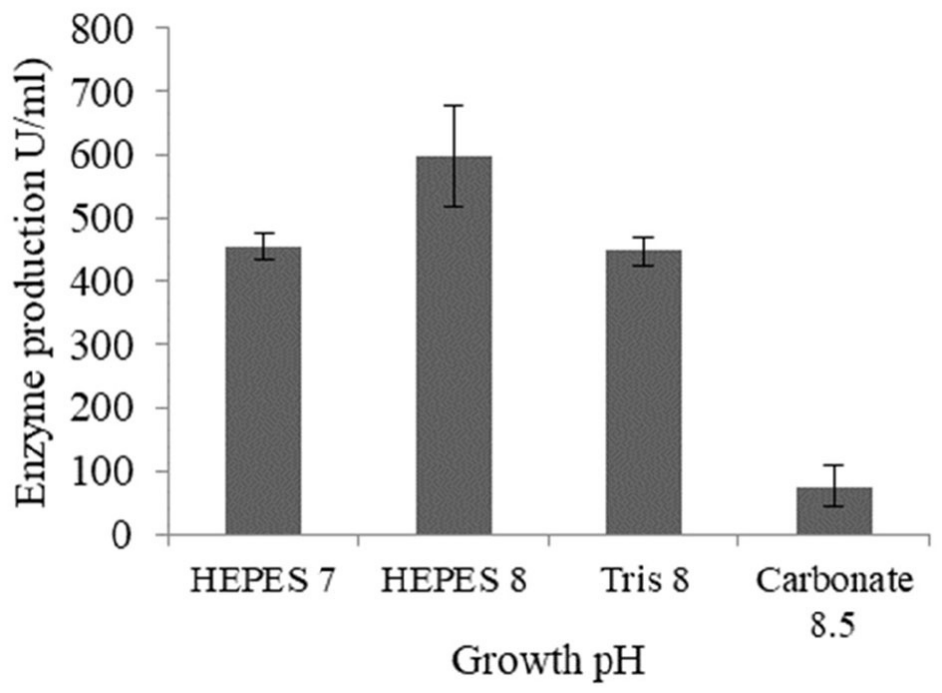
Effect of pH and buffer type on lipase production from *Brevibacillus* sp. SHI-160. The pH of the medium was adjusted using different buffers prior to inoculation. The buffers used were HEPES (pH 7.0), HEPES (pH 8.0), Tris-HCl (pH 8.0), and carbonate bicarbonate (pH 8.5).

Lipase production by *Brevibacillus* sp. SHI-160 was highest when the medium pH was adjusted to 8.0 using HEPES buffer (Figure 3). A lower level of lipase production was observed when the medium pH was adjusted to the same value (pH 8.0) using Tris-HCl buffer, indicating that, in addition to the pH value, the buffer used could also be important in determining enzyme production. A significant decrease in lipase production was observed in pH 8.5 carbonate bicarbonate buffer (Figure 3).

#### 3.2.3. Time course of production

**Figure 4:**
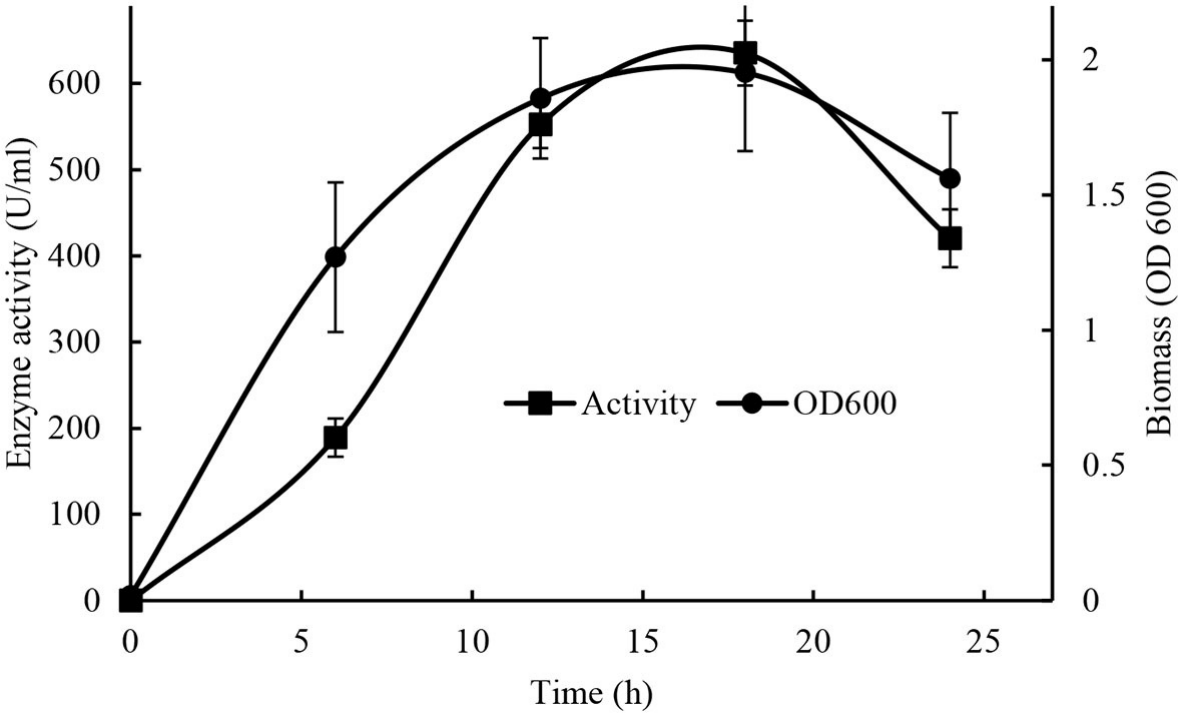
Effect of production time on cell growth and maximum lipase production (Cell growth •) and (activity ■).

The highest production of lipase by *Brevibacillus* sp. SHI-160 (653 U/ml) was obtained after 18 h of cultivation in a basal medium containing 0.25% yeast extract and 0.5% glucose at pH 8 and 55°C (Figure 4). The time at which the highest lipase production reached corresponds to the stationary phase of growth of the microorganism. Lipase production was already detectable after 6 h of incubation, and after 18 h, both growth and enzyme production started to decline, indicating cell death.

### 3.3. Purification and characterization of *Brevibacillus* sp. SHI-160 lipase

**Figure 5:**
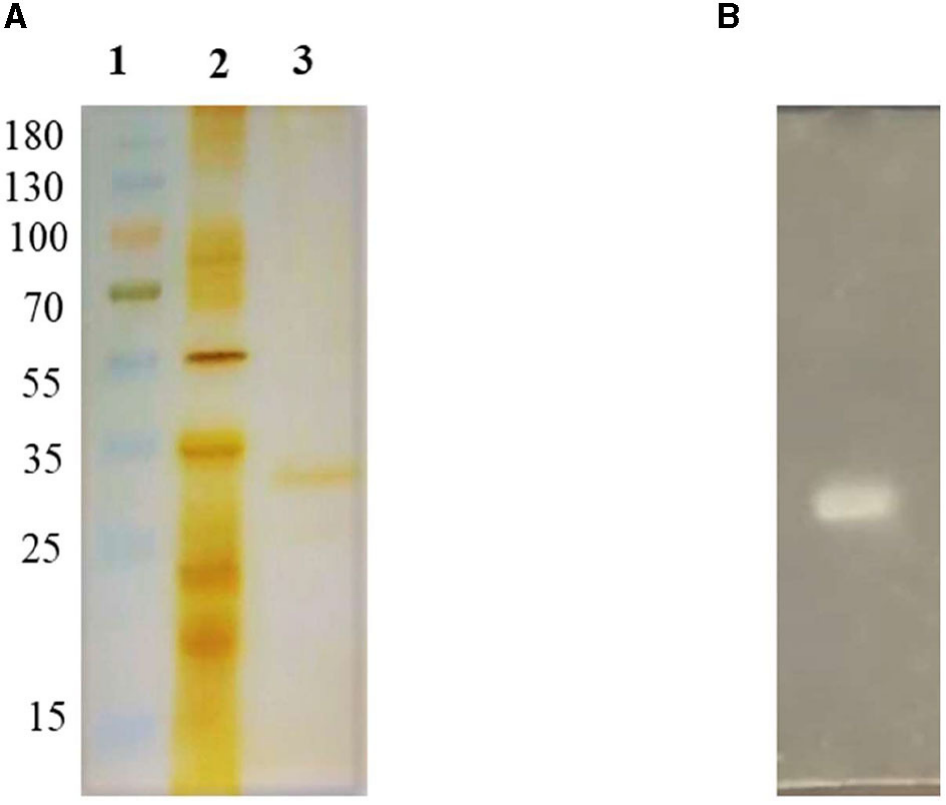
SDS PAGE **(A)** and zymogram **(B)** of *Brevibacillus* sp. SHI-160 lipase. Lane 1: Markers; Lane 2: ammonium sulfate concentrated preparation; and Lane 3: purified lipase.

*Brevibacillus* sp. SHI-160 lipase was purified following ammonium sulfate precipitation and ion exchange chromatography. The enzyme has an estimated molecular weight of 33.5 kDa (Figure 5A) and a specific activity of 54,404 U/mg (Supplementary Table 3). Zymography using Tween 80 hydrolysis showed the presence of only one lipase (Figure 5B).

#### 3.3.1. Effect of temperature on the activity and stability

**Figure 6:**
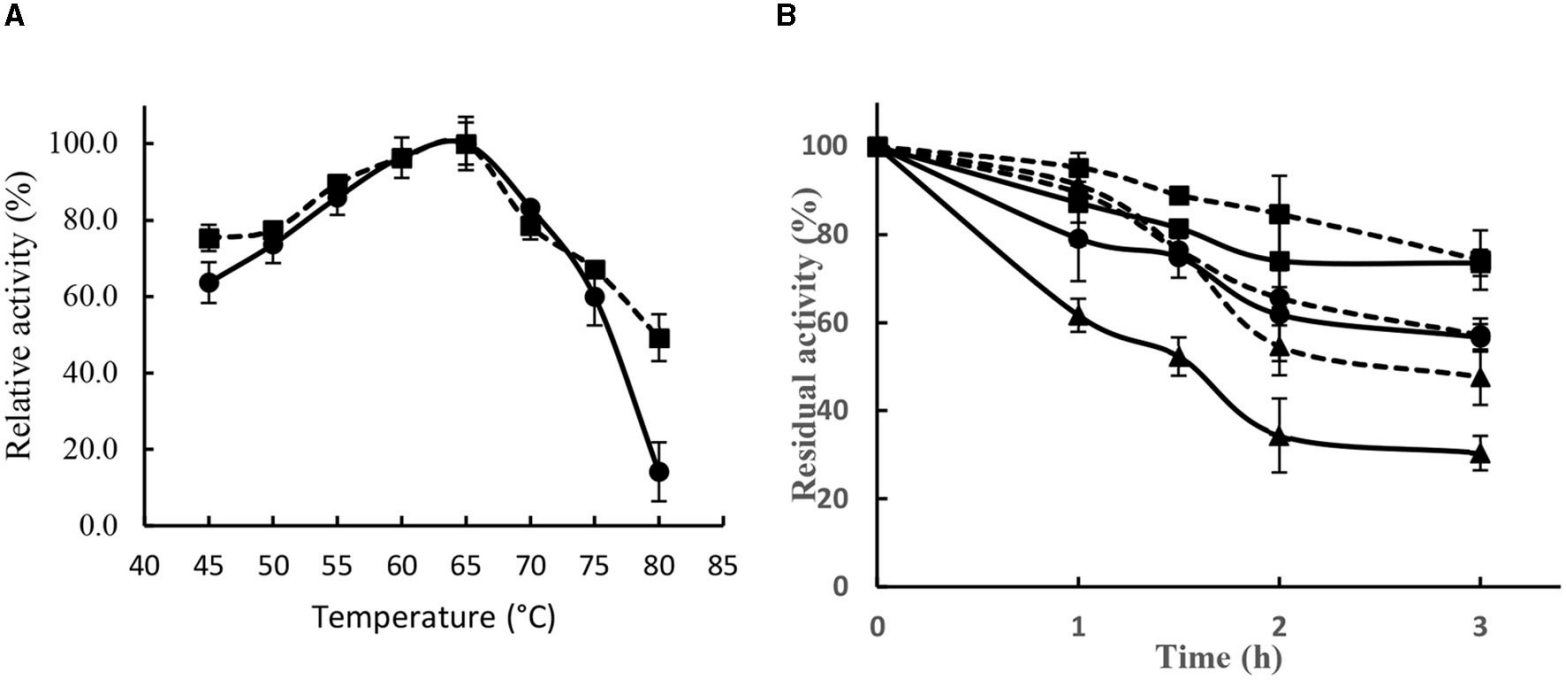
Effect of temperature on the activity **(A)** and stability **(B)** of *Brevibacillus* sp. SHI-16 lipase. Enzyme activity was assayed in the presence (■) and absence (•) of calcium ions. Stability was determined at 60°C (■), 65°C (•), and 70°C (▴) in the presence (dotted lines) and absence (solid lines) of Ca^2+^.

Lipase SHI-160 showed its highest activity at 65°C and retained ~80% of that activity in the temperature range of 55°C and 70°C (Figure 6A). The addition of 5 mM calcium ion in the assay buffer had no effect on enzyme activity in temperature ranges below 75°C. However, at 80°C, a marked difference in activity was observed in the presence and absence of calcium (Figure 6A).

Lipase SHI-160 showed good stability in the temperature range of 60 to 70°C (Figure 6B). In the presence of calcium, after 1 h of incubation at 60, 65, and 70°C, the enzyme retained over 90% of its original activity. After 3 h of incubation, the enzyme retained 74, 57, and 48% of its original activity, respectively (Figure 6B). However, in the absence of calcium, enzyme stability decreased with increasing temperature, indicating that the enzyme requires calcium for stability (Figure 6B).

#### 3.3.2. Effect of pH on the activity and stability

**Figure 7:**
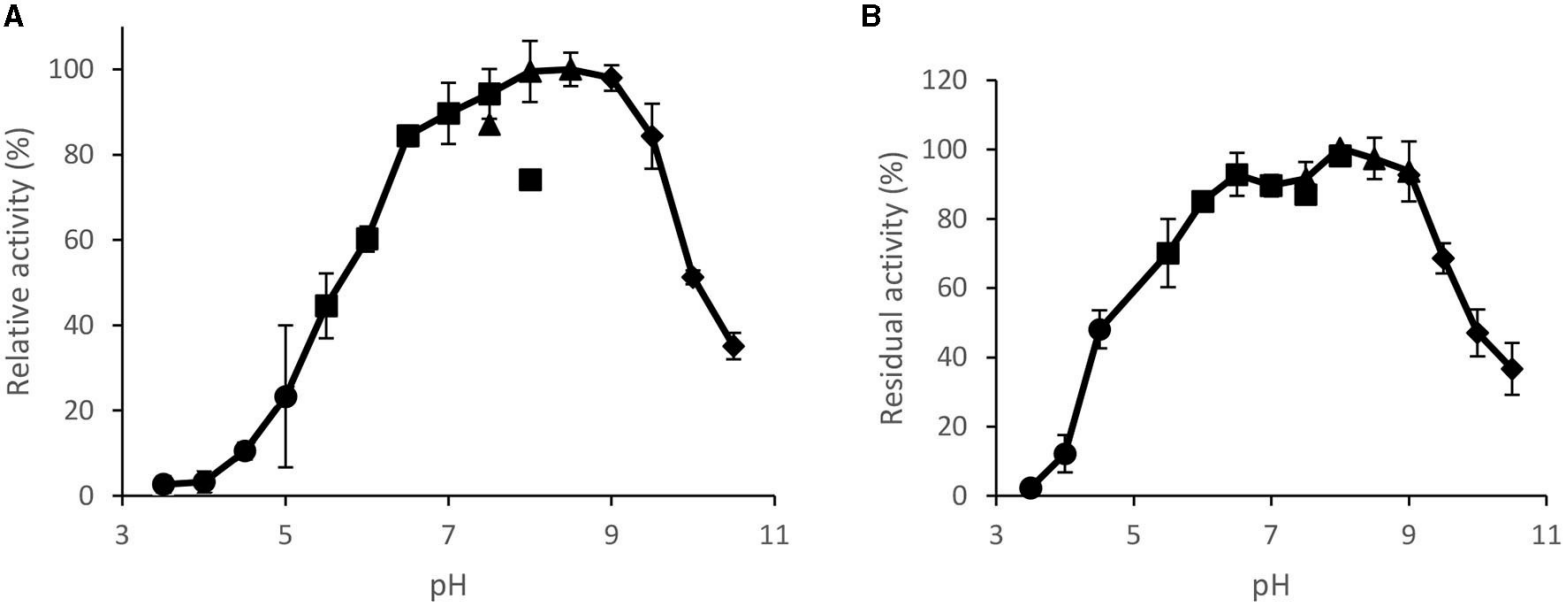
Effect pH on purified *Brevibacillus* sp. SHI-160 lipase **(A)** activity and **(B)** stability. Lipase incubated for 1 h at RT in different buffers and assays at 65°C and 20 mM of different buffers within 5 min of lipase assay. Buffers used (each at 50 mM) were acetate (pH 3.5–5.5), phosphate (pH 5.5–8.0), Tris-HCl (pH 7.5–9.0), and glycine-NaOH (pH 9.0–10.5).

The enzyme was active in a broad range of pH ranging from 6.5 to 9.5, with the highest activity at pH 8.5 (Figure 7A). At pH 10, the enzyme displayed ~50% of its maximum activity. The enzyme was also stable in a broad range of pH retaining more than 90% of its original activity after 1 h of incubation in pH between 6.5 and 9.0 (Figure 7B).

#### 3.3.3. Effect of metal ions and inhibitors on the stability

At concentrations of 1 mM and 5 mM, the metal ions Mg^2+^, Co^2+^, Ca^2+^, K^+^, Mn^+^, and Na^+^ did not have any significant effect on the lipase SHI-160, but the enzyme was inhibited to different degrees in the presence of Fe^+^, Zn^2+^, and Cu^2+^ (Table 2).

**Table 2:** Effect of different metal ions on the stability of lipase after 1 h of incubation at 37°C.

**Metal ion**	**Residual activity (%)**	
	**1 mM**	**5 mM**
Mn^2+^	84.5 ± 12	99.6 ± 6.1
Co^2+^	92.3 ± 4.8	92.3 ± 4.4
Mg^2+^	97.6 ± 11.5	82.7 ± 3.1
Na^+^	87.2 ± 8.6	83.0 ± 10.0
Ca^2+^	108.1 ± 2.1	109.8 ± 12.6
Cu^2+^	70.4 ± 0.4	25.5 ± 7.7
Fe^2+^	51.3 ± 4.2	29.4 ± 1.5
K^+^	101.0 ± 5.8	92.3 ± 16.4
Zn^2+^	70.6 ± 14.9	57.0 ± 8.5
PMSF	105.0 ± 11.6	92.1 ± 8.5
SDS	105.8 ± 9.7	24.7 ± 3.8
EDTA	79.3 ± 11.3	54.1 ± 7.0
DTT	92.8 ± 13.4	79.1 ± 2.7

The enzyme retained its original activity in the presence of 1 mM PMSF, DTT, and SDS, while an increase in the concentration of SDS to 5 mM resulted in a decrease in the enzyme activity by 75% (Table 2).

#### 3.3.4. Effect of organic solvent on the stability

Lipase SHI-160 was stable in the presence of different water-miscible and water-immiscible organic solvents at a concentration of 25 and 50% (Table 3). Even in the presence of 75% solvent, lipase SHI-160 retained more than 50% of its original activity in the presence of most of the solvents tested. However, no activity was detected after 1 h of incubation in the presence of 75% DMSO and ethyl acetate (Table 3). The stability of SHI-lipase in polar organic solvents was better than most organic solvent-tolerant lipases and esterases (Supplementary Table 4) (Perez et al., 2011; Samaei-Nouroozi et al., 2015: Rmili et al., 2019; Singh et al., 2019; Park et al., 2020).

**Table 3:** Effect of various organic solvents on the stability of purified lipase form *Brevibacillus* sp. SHI-160 after 1 h of incubation at 37°C.

**Solvent**	**Residual activity (%)**		
	**25%**	**50%**	**75%**
Acetone	88.1 ± 3.6	71.4 ± 6.4	61.9 ± 3.7
Ethanol	114.8 ± 0.9	97.9 ± 3.2	63.8 ± 6.7
Methanol	105.6 ± 0.7	100.3 ± 5.2	66.4 ± 1.4
DMSO	88.1 ± 3.6	102.1 ± 0.9	0
Isopropanol	118.5± 1.6	104.2 ± 4.8	68.6 ± 11.4
Hexane	105.3 ± 7.2	63.3 ± 10.7	ND
Ethyl acetate	124.9 ± 7.1	52.2 ± 12.4	0
Acetonitrile	118.3 ± 12.8	87.0 ± 6.1	53.2 ± 9.7

#### 3.3.5. Effect of NaCl concentration on the activity and thermal stability of lipase

When assayed in the presence of different salt concentrations, lipase SHI-160 retained 100% of its original activity or showed slight enhancement of activity until the salt concentration increased to ~1M. When the salt concentration was doubled to 2 M, the enzyme retained ~70% of its original activity (Supplementary Figure 2).

### 3.4. Partitioning of lipase SHI-160 using alcohol-salt ATPS

#### 3.4.1. Determination of the bimodal curve and lipase portioning using isopropanol-salt ATPS

The bimodal cure for isopropanol and potassium phosphate salt was determined by mixing different concentrations of the alcohol and the salt (Supplementary Figure 3). An isopropanol concentration of 16–24 % (w/w) and a potassium phosphate concentration of 16–24% (w/w) were selected for optimization for the recovery of lipase directly from *Brevibacillus* sp. SHI-160 culture without prior cell separation through centrifugation.

When the whole culture (without cell separation) was applied directly to an isopropanol-salt ATPS, each in the concentrations range of 16 to 24% (w/w), between 52.57 and 92.67% of the lipase partitioned to the top alcohol phase. The highest partitioning of lipase SHI-160 of 92.67% was obtained at alcohol and salt concentrations of 16% (w/w) each (16/16) (Table 4). At this concentration, lipase SHI-160 had a partition coefficient of (Ke) of 28.05 and a purification level of 4.94-fold. As the alcohol and salt concentrations increased beyond 16/16 (%, w/w), both the partition coefficient and lipase recovery decreased (Table 4).

**Table 4:** Extraction of lipase in isopropanol/phosphate ATPS system at 25°C pH 8 in 5 g system.

**Isopropanol (%, w/w)**	**Potassium salt (%w/w)**	**Ke**	**Specific activity (U/μg)**	**Kp**	**S**	**Purification fold**	**Yield (%)**
16	16	28.05 ± 0.2	9.48 ± 8.17	0.2 ± 0.001	121.58 ± 1.7	4.94 ± 0.004	92.7 ± 0.08
	18	21.1 ± 0.19	8.43 ± 3.4	0.2 ± 0.001	86.2 ± 0.29	4.4 ± 0.00	86.7 ± 0.8
	21	13.8 ± 0.88	5.024 ± 1.99	0.48 ± 0.01	29 ±0.31	2.62 ± 0.00	84.5 ± 0.66
	24	4.1 ± 0.1	3.66 ± 14.1	0.5 ± 0.0	8.0 ± 0.0	1.9 ± 0.0	65.2 ± 0.8
18	16	11.3 ± 0.17	8.22 ± 16.78	0.3 ± 0.00	42.5 ± 0.89	4.3 ± 0.00	89.8 ± 0.18
	18	9.72 ± 0.29	5.61 ± 7.4	0.41 ± 0.04	23.4 ± 1.3	2.9 ± 0.004	82.7 ± 1.2
	21	4.8 ± 0.101	4.32 ± 32.045	0.2 ± 0.337	10.0 ± 0.252	2.3 ± 0.017	72.6 ± 0.740
	24	3.2 ± 0.05	3.314 ± 5.06	0.5 ± 0.02	6.0 ± 0.075	1.7 ± 0.003	59.9 ± 0.958
21	16	10.0 ± 0.118	6.18 ± 11.101	0.4 ± 0.01	27.0 ± 0.815	3.2 ± 0.006	87.6 ± 1.268
	18	9.4 ± 0.448	4.64 ± 7.96	0.5 ± 0.01	18.59 ± 1.2	2.418 ± 0.004	81.58 ± 1.1
	21	4.4 ± 0.007	4.343 ± 7.86	0.5 ± 0.005	8.08 ± 0.068	2.26 ± 0.004	79.85 ± 0.37
	24	3.74 ± 0.29	3.215 ± 3.2	0.54 ± 0.03	6.88 ± 0.11	1.68 ± 0.002	58.69 ± 2.5
24	16	7.67 ± 0.34	5.00 ± 0.06	0.48 ± 0.02	15.8 ± 0.01	2.61 ± 0.001	84.62 ± 2
	18	6.7 ± 0.526	4.746 ± 31.285	0.5 ± 0.015	13.9 ± 0.659	2.5 ± 0.016	78.7 ± 3.993
	21	3.8 ± 0.333	4.65 ± 91.371	0.4 ± 0.002	9.7 ± 0.898	2.4 ± 0.048	68.4 ± 1.085
	24	2.86 ± 0.37	3.2 ± 4.7	0.53 ± 0.02	5.40 ± 0.86	1.65 ± 0.00	52.57 ± 0.7

#### 3.4.2. Lipase partitioning in the presence of NaCl

While lipase recovery using the 16/16 (%, w/w) isopropanol-salt ATPS was ~92.2%, the addition of 1, 2, and 3% NaCl to the above mixture increased lipase recovery to 96.83, 98.17, and 95.86%, respectively. Increasing NaCl concentration over 2% resulted in a decrease in lipase recovery. Upon addition of NaCl, the partition coefficient increased from 31.3% in the absence of salt to 67.02% in the presence of 2% salt (Table 5).

**Table 5:** Effect of NaCl on the portioning of lipase in isopropanol/potassium phosphate ATPS at pH 8 and 25°C.

**NaCl (%, w/w)**	**Ke**	**Specific activity U/μg**	**Kp**	**S**	**PF (t) = SAt/**	**Yield (%)**
0	31.3 ± 1.793	14.9 ± 1.07	0.27 ± 0.013	113.57 ± 1.04	6.54 ± 0.47	92.2 ± 1.0
1	36.6 ± 0.76	16.69 ± 8.33	0.24 ± 0.01	152.43 ± 8.25	7.34 ± 0.24	96.83 ± 0.45
2	67.02 ± 0.49	19.07 ± 1.5	0.43 ± 0.031	153.4 ± 12.26	8.39 ± 0.64	98.17 ± 0.43
3	37.3 ± 0.1	16.87 ± 0.21	0.31 ± 0.014	119.5 ± 5.3	7.43 ± 0.094	95.86 ± 0.935
4	17.17 ± 0.31	13.7 ± 1.55	0.26 ± 0.036	65.1 ± 7.744	6.04 ± 0.68	87.23 ± 1.3
5	14.8 ± 0.7	13.2 ± 0.07	0.27 ± 0.003	55.37 ± 3.56	5.82 ± 0.033	76.5 ± 0.25

#### 3.4.3. Immobilization of ATPS recovered lipase and test for transesterification reaction

**Figure 8:**
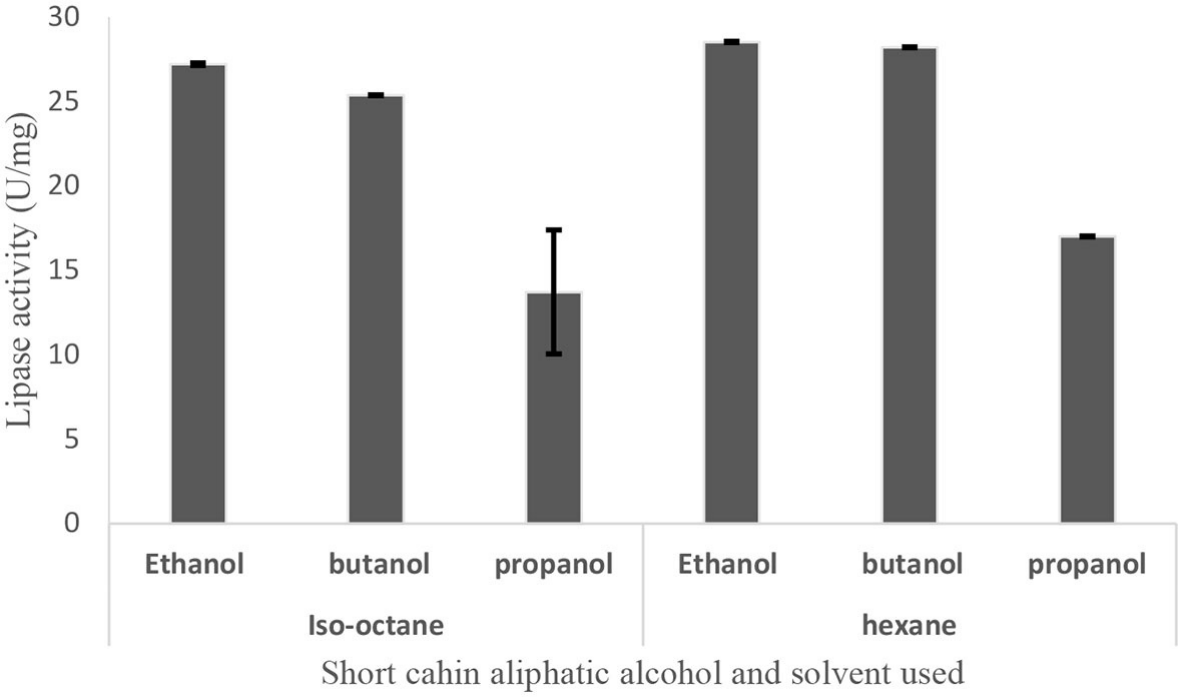
Transesterification of PNPP with short-chain alcohol using hexane and isooctane as a solvent at 65°C for 30 min.

Upon direct adsorption of alcohol-salt ATPS recovered lipase on celite followed by drying at 45°C, 91.9% of the lipase activity of the original culture was successfully immobilized (Table 6). The resulting immobilized dry enzyme preparation efficiently transesterified pNPP with three different short-chain alcohols in the presence of hexane or isooctane as solvents. The transesterification reaction was highest when ethanol and butanol were used as substrates (Figure 8).

**Table 6:** Adsorption of ATPS recovered lipase onto celite.

**Sample**	**Volume/weight**	**Activity**	**Total activity (U)**	**Yield (%)**
Culture	110 ml	442.81 ± 0.36 U/ml	48,709.1 ± 39.4	100
ATPS	64.4 ml	712.9 ± 0.5 U/ml	47,335.3 ± 35.7	97.1 ± 0.07
Immobilized lipase	1.5 g	29.9 ± 0.023 U/mg	44,814.7 ± 35.0	91.9 ± 0.07

## 4. Discussion

In this study, *Brevibacillus* sp. SHI-160, a lipase-producing moderately thermophilic strain, was isolated from a hot spring in the central Great Rift Valley of Ethiopia. The enzyme exhibited good activity and stability at elevated temperature and in the presence of polar and non-polar organic solvents, indicating its potential applications to catalyze reactions in non-aqueous media and at elevated temperature. Optimum growth and maximum enzyme production were observed at pH 8.0, which corresponds to the pH range of 8.0–8.9 of the hot spring from where the organism was isolated. Previously, different thermostable microbial enzymes of potential industrial importance were isolated from high-temperature environments in the Ethiopian Rift Valley (Gessesse, 1998; Mamo and Gessesse, 1999; Gulelat and Tilahun, 2012). However, to the best of our knowledge, this is the first microbial lipase to be reported from a strain isolated from this unique habitat. The isolation of the lipase-producing strain, *Brevibacillus* sp. SHI-160, and the other microbial strains isolated previously indicate the potential of the microbial genetic resource of the extreme environments in the Great East African Rift Valley as a source of novel lipases and other industrially important enzymes.

The amount and type of nitrogen source are known to have a significant influence on the growth and production of lipase (Adetunji and Olaniran, 2021; Fatima et al., 2021). Peptone and yeast extract, often used in combination, are the most common organic nitrogen sources used for lipase production. In this study, the highest lipase production by *Brevibacillus* sp. SHI-160 was achieved when grown in the presence of yeast extract as the sole nitrogen source. Similar reports of better lipase production using yeast extract alone were reported for *Bacillus amyloliquefaciens* (Mazhar et al., 2023), *Burkholderia multivorans* (Gupta et al., 2007), *Trichosporon asahii* MSR 54 (Kumar and Gupta, 2008), and *Pseudomonas aeruginosa* (Ilesanmi et al., 2020). Since yeast extract is rich in vitamins, peptides, amino acids, phosphate, and other essential minerals (Li et al., 2011; Tomé, 2021; Tao et al., 2023), it provides the nutritional requirements of the organism for optimum growth and enzyme production.

*Brevibacillus* sp. SHI-160 produced an appreciable level of enzyme activity when grown in the presence of sugar and in the absence of any triglyceride, indicating that lipase production is constitutive. Although the organism produced lipase in the presence of either sugar sources or olive oil as carbon sources, up to more than 3-fold lipase was produced when glucose was used as the sole carbon source. A high level of lipase production was also observed when other sugars such as sucrose, xylose, or glycerol were used. The addition of olive oil to the medium containing the above sugars did not result in an increase in enzyme production. Although there are reports on constitutive lipase production by some microbial strains (Gupta et al., 2004), most known microbial lipases are inducible, requiring the addition of triglycerides as inducers (Salihu et al., 2012; Zarevúcka, 2012). For *Brevibacillus* sp. SHI-160, the addition of inducers such as plant oils and Tween 80 resulted in a decrease rather than an increase in lipase production. A similar observation of lower extracellular lipase production in the presence of lipid substrates, especially in the presence of olive oil in the medium containing different carbohydrates, was previously reported for *Bacillus licheniformis* PAL05 (Anbu and Hur, 2014), *Nocardiopsis* sp. (Aziz et al., 2020), and *Bacillus stearothermophilus* (Kambourova et al., 2003). On the other hand, for *Candida rugosa* (Dalmau et al., 2000) and *Pseudomonas aeruginosa* (Ilesanmi et al., 2020), the presence of glucose in the culture medium resulted in the repression of lipase production. From a practical point of view, constitutive lipase production by *Brevibacillus* sp. SHI-160 in the absence of any lipid substrate could help to avoid any residual lipid that could interfere during downstream processing and thus help in lowering enzyme production cost.

Having a molecular weight of 33.5 kDa, lipase SHI-160 is of similar molecular size to most microbial lipases that fall in the range of 19 to 69 kDa. Some of the reported molecular weights of microbial lipases include 31 kDa for *Bacillus methylotrophicus* PS3 (Sharma et al., 2017), 35 kDa for *Bacillus licheniformis* strain SCD11501 (Sharma et al., 2017), 43 kDa for *Staphylococcus aureus* (Bacha et al., 2018), 50 kDa for *Geobacillus thermodenitrificans* AV-5 (Christopher et al., 2015), and 67 kDa for *Bacillus stearothermophilus* (Bacha et al., 2015).

Lipases from most organisms are active and stable around ambient temperature. On the other hand, many industrial reactions involving lipases are carried out at high temperature, requiring the use of active and stable enzymes at elevated temperature (Hasan et al., 2006; López-López et al., 2014; Mo et al., 2016; Vivek et al., 2022; Akram et al., 2023; Ali et al., 2023). Lipase SHI-160 exhibited optimum activity at 65°C and retained 90% of its activity after 1 h of incubation at 70°C in the presence of Ca^2+^, indicating the contribution of this metal ion to stabilizing the enzyme. Most lipases and many other extracellular microbial enzymes are known to require Ca^2+^ to maintain the conformational stability of the proteins (Hertadi and Widhyastuti, 2015; Ishak et al., 2019).

The enzyme was also active in a broad pH range (between 6.5 and 9.5) with a peak activity at pH 8.5, indicating its potential use to catalyze reactions in the alkaline range. To date, only a few microbial strains such as *Geobacillus thermodenitrificans* AV-5 (Christopher et al., 2015) and *Streptomyces* sp. CS268 (Mander et al., 2012) were reported to produce thermostable lipases with optimum activity in the pH range of 8.0–9.0. The molecular structure of enzymes is maintained by non-covalent bond interactions of amino acid side chains, many of which are affected by the pH of the medium. Therefore, deviation of pH away from its optimum can lead to an ionization state of amino acid side chains and disrupt the bonds that maintain the 3D structure of protein molecules.

Due to the advantages of the process, most industrial reactions involving lipases are carried out in non-aqueous media (Ismail et al., 2021; Patel and Parikh, 2022; Akram et al., 2023). Therefore, in addition to stability and activity at elevated temperature and extreme pH values, activity and stability in the presence of organic solvents are essential properties for the industrial application of lipases (Kumar et al., 2016; Haryati et al., 2022; Ali et al., 2023). Lipase SHI-160 was stable in the presence of different polar and non-polar organic solvents. Although lipases stable in non-polar organic solvents are relatively common (Kumar et al., 2016; Ismail et al., 2021), stability and activity in the presence of polar organic solvents are rare (Sardessai and Bhosle, 2004; Ishak et al., 2019; Vahidi et al., 2021). Therefore, the stability and activity of lipase SHI-160 in the presence of polar organic solvents, such as ethanol, methanol, acetone, and isopropanol, makes it attractive for use in reactions.

Halotolerant enzymes, mostly derived from halophiles, are generally considered tolerant to organic solvents because of their adaptation to function under low water activity (Li and Yu, 2014; Kikani et al., 2023). For example, halotolerant lipase from *Halomonas* sp. C2SS100 retained its full activity in the presence of 10 g/l (0.17 M) NaCl, but its activity dropped to ~40% upon increasing the salt concentration to 50 g/l (0.85 M) (Khmaissa et al., 2022). In this study, lipase SHI-160 displayed 100% activity in the presence of 1 M NaCl (58.5 g/l) and retained over 60% of its activity in the presence of 2 M salt (117 g/l), indicating its salt tolerance, which also corresponds to its tolerance to organic solvents. On the other hand, the stability of lipase SHI-160 was reduced in the presence of Cu^2+^, Fe^2+^, and Zn^+^. A reduction in stability in the presence of the above three metal ions was also reported for other thermostable lipases (Sharma et al., 2017). After the addition of EDTA, a reduction in stability was also observed, especially at higher concentration. Since the enzyme required calcium for stability at high temperature, the addition of EDTA might have led to the removal of the required Ca^2+^ ion, compromising its stability. The enzyme was also stable in the presence of 1 mM SDS but lost ~75% of its activity when the concentration of the detergent increased to 5 mM, indicating denaturation. Some thermostable lipases were reported to show good stability in the presence of up to 10% SDS (Lianghua and Liming, 2005).

The stability of lipase SHI-160 in the presence of polar organic solvent, such as isopropanol, allowed the development of an alcohol/salt-based aqueous two-phase system for the recovery of the enzyme directly from the culture without a need for centrifugation or enzyme precipitation. Up to 98% of the lipase activity in the culture was recovered using the alcohol/salt ATPS. Because the enzyme is stable in the presence of polar organic solvents, there was no loss of activity upon exposure to the alcohol-rich phase. As the concentration of potassium phosphate increased beyond the optimum, a reduction in lipase recovery was observed probably due to the formation of protein aggregates, as reported earlier (Xu et al., 2015). Partitioning of the lipase to the alcohol phase significantly improved upon addition of 2% NaCl probably due to the generation of electrical potential difference between the top phases as proposed earlier (Phong et al., 2018). Despite the growing interest to use microbial lipases for different applications, the enzyme is often expensive, limiting its wider application in different industrial processes. A high downstream processing cost has a significant contribution to the overall production cost of the enzyme (Chandra et al., 2020). Therefore, the development of cheap and simple downstream processing of lipase SHI-160 using an alcohol/salt ATPS could help to reduce the enzyme production cost and increase its potential application in different industrial processes.

After lipase partition to the alcohol phase, subsequent enzyme formulation in dry powder form requires the separation of protein from the alcohol, which could lead to an additional processing step. In this study, the enzyme, while still suspended in the alcohol phase, was immobilized by adsorption onto Celite 545^TM^, resulting in the immobilization of over 91% of the original lipase activity. The dry immobilized enzyme was then successfully used to catalyze a transesterification reaction between pNPP and short-chain alcohols in non-aqueous media. At the end of the fermentation process, no centrifugation or filtration was required to separate the crude enzyme from the cell biomass. In addition, there was also no need for enzyme precipitation or concentration before the immobilization step. Therefore, direct recovery of the enzyme from the culture through an alcohol/salt-based ATPS and direct immobilization of the enzyme from the alcohol phase could allow a significant reduction in enzyme production cost.

## Data availability statement

The datasets presented in this study can be found in online repositories. The names of the repository/repositories and accession number(s) can be found in the article/Supplementary material.

## Author contributions

SL: Formal analysis, Methodology, Writing—original draft, Writing—review and editing, Data curation, Investigation, Visualization. EJ: Formal analysis, Methodology, Writing—review and editing, Conceptualization, Funding acquisition, Project administration, Resources, Supervision, Validation. RV: Formal analysis, Methodology, Supervision, Writing—review and editing, Investigation. EC: Formal analysis, Methodology, Supervision, Writing—review and editing. AG: Formal analysis, Methodology, Supervision, Writing—review and editing, Conceptualization, Funding acquisition, Investigation, Project administration, Resources, Validation, Writing—original draft.
