# Supplementary material for: A thermostable organic solvent-tolerant lipase from Brevibacillus sp.: production and integrated downstream processing using an alcohol-salt-based aqueous two-phase system
Source: Front Microbiol. 2023 Oct 13;14:1270270. doi: 10.3389/fmicb.2023.1270270 (PMC10612343; doi:10.3389/fmicb.2023.1270270)
Supplement: Supplementary file 1 [file Data_Sheet_1.docx]

Supplementary Material


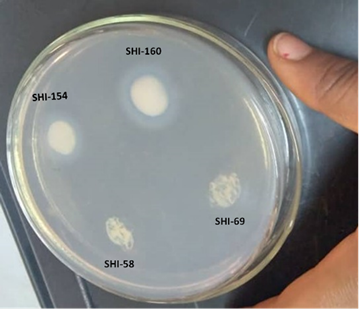


Supplementary Fig. 1


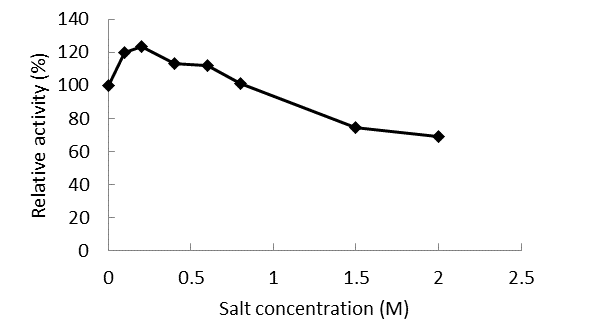


Supplementary Fig. 2. (Supp)

Supplementary Fig. 3

**Legends to Figures**

Supplementary Fig. 1. Screening for lipase production using Tween-80 agar plates. Lipase positive isolates form a white precipitate around the colony (SHI-160 and SHI-154).

Supplementary Fig. 2. Effect of NaCl concentration on the activity of purified lipase from *Brevibacillus* SHI-160.

Supplementary Fig. 3. Bimodal curve of Isopropanol/Potassium phosphate at pH 8, at 25^0^C

**List of supplementary Tables**

Supplementary Table 1: Parameters for the evaluation of lipase recovery using salt aqueous two-phase system (ATPS).

| Ke* | Kp | Specific activity(U/µg) | Purification fold | Selectivity | Yield (%) |
| --- | --- | --- | --- | --- | --- |
| $\frac{\boldsymbol{LA} \boldsymbol{t}}{\boldsymbol{LA} \boldsymbol{b}}$ | $\frac{Pt}{Pb}$ | $\frac{\mathrm{LAt}}{\mathrm{Pt}}$ | $\frac{\mathrm{SAt}}{\mathrm{SAc}}$ | $\frac{\mathrm{Ke}}{\mathrm{KP}}$ | $100-\frac{(LAc-LAt)}{\mathrm{LAc}})*100$ |

* Ke, Kp represents partition coefficient of lipase and protein respectively. LAt, LAb and LAc, denotes lipase activity of top, bottom crude; whereas Pt and Pb; denotes total protein top and bottom, SAc and SAt represents lipase specific activity of crude and top phase.

Supplementary Table 2. Effect of different lipids on growth of *Brevibacillus* sp. SHI-160.

| Carbon source* | OD 600 | Relative growth Reduction (%) |
| --- | --- | --- |
| Tween 80 + glucose | 0.436 | 52.3 |
| Sunflower oil + glucose | 0.63 | 31.1 |
| Olive oil + glucose | 0.799 | 12.7 |
| Glucose | 0.915 | 0.0 |

Supplementary Table 3. Purification table of SHI-160 lipase by *Brevibacillus* sp. SHI-160.

| Sample | Total protein (mg) | Total activity (U) | Specific activity (U/mg) | Yield (%) | Purification fold |
| --- | --- | --- | --- | --- | --- |
| Crude | 86.74 | 137,419 | 1,584 | 100 | 1 |
| Ammonium sulphate | 5.91 | 46,543 | 7,867 | 34 | 5 |
| DEAE cellulose | 0.57 | 31,119 | 54,404 | 23 | 34 |

Supplementary Table 4. Comparison of organic solvent tolerance of different lipases in different with *Brevibacillus* sp. SHI-160 lipase.

| Enzyme (source organism) | Test condition (temperature & time) | Stability in organic solvents (% residual activity) | Reference |
| --- | --- | --- | --- |
| Lipase *(Brevibacillus* sp. SHI-160) | 37°C for 1 h in aqueous mixture | 97.9 % in 50% ethanol  100.3 % in 50% methanol  87% in 50% acetonitrile  118.3% in 25% acetonitrile  71.4 % in 50% acetone  105.3% in 25% hexane  63% in 50% hexane  124.9% in 25% ethyl acetate  52.2% in 50% ethyl acetate | This study |
| Lipase *(Alkalibacillus salilacus)* | 40°C for 1 h in aqueous mixture | 21% in 50% acetonitrile  29% in 50% butanol  50% in 50% acetone  83% in 50% hexane  85% in 50% ethanol | Samaei-Nouroozi et al., 2015 |
| Lipase *(*Marinobacter lipolyticus SM19) | 25°C for 30 min in aqueous mixture | 94% in 5% hexane  27% in 30% acetonitrile  120% in 30% methanol  35% in 30% 1-propanol | Perez et al., 2011 |
| Lipase *(Bacillus* sp. DM9K3) | 50°C for 4 h in in aqueous mixture | 50 – 60% in 33% acetone  40 – 50% in 33% ethanol  100- 110% in 33% hexane  100% in 33% toluene | Singh et al., 2019 |
| Lipase *(*Geobacillus zalihae HT1  Wildtype enzyme) | 60°C, 30 min in aqueous mixture | <5% in 25% 1-propanol  20-25% in 25% methanol  20 – 25% in 25% acetonitrile  <40% in 25% ethanol  100% in 25% hexane  100% in 25% DMSO | Ishak et al., 2019 |
| Lipase *(Staphylococcus capitis)* | 4°C, 30 min in aqueous mixture | ~60% in 15% ethanol  ~70% in 15% methanol  ~75% in 15% acetonitrile  ~90% in 15% acetone  ~95% in 15% isopropanol | Rmili et al., 2019 |
| Lipase *(Pseudomonas fluorescens)* | 40°C, 30 min in aqueous mixture | 45.79% in 30% ethanol  76.45% in 30% Isopropanol  86.56% in 30% DMSO  91.18% in 30% acetonitrile  92.57% in 30% hexane | Hu et al., 2023 |
| Lipase *(Streptomyces pratensis* MV1) | 25°C, 1 h in aqueous mixture | 35.3% in 25% acetonitrile  0% in 50% acetonitrile  27.9% in 50% ethyl acetate  59.6% in 50% DMSO  84% in 25% methanol  69.8% in 50% methanol  121% in 50% hexane | Vahidi et al., 2021 |
| Esterase  (from compost metagenomic library) | 30°C, 1 h in aqueous mixture | 25.2% in 50% 2-propanol  115.8% in 30% 2-propanol  43.0% in 50% acetonitrile  101.1% in 30% acetonitrile  43.4% in 50% methanol  50.3% in 50% ethanol  101.4% in 30% ethanol  66.1% in 50% DMSO  74.4% in 50% DMSO  81.8% in 50% acetone  100% in 30% acetone | Park et al., 2020 |
| Esterase  (*Salimicrobium*sp. LY19) | 30°C, 1 h in aqueous mixture | 11.9% in 20% acetonitrile  15.5% in 20% ethanol  17.3% in 20% acetone  18.4% in 20% methanol  81.4% in 20% DMSO  86.6% in 20% n-hexane | Xin and Hui-Ying, 2013 |
